# Experiences of an interprofessional follow-up program in primary care practice

**DOI:** 10.1186/s12913-024-10706-9

**Published:** 2024-02-23

**Authors:** Beate-Christin Hope Kolltveit, Bjørg Frøysland Oftedal, Sally Thorne, Kirsten Lomborg, Marit Graue

**Affiliations:** 1 Department of Health and Caring Sciences, Western Norway University of Applied Sciences, Bergen, Norway; 2 Faculty of Health Sciences, University of Stavanger, Stavanger, Norway; 3 School of Nursing, University of British Columbia, Vancouver, CA Canada; 4 Vossevangen medical centre, Voss, Norway; 5 Department of Clinical Medicine, University of Copenhagen, Copenhagen, Denmark; 6 Department of Clinical Research, Copenhagen University Hospital - Steno Diabetes Center Copenhagen, Copenhagen, Denmark

**Keywords:** Qualitative, Interpretive description, Primary care, Interprofessional collaboration

## Abstract

**Background:**

An integrative cooperation of different healthcare professional is a key component for high quality health services. With an aging population and many with long-term conditions, more health tasks and follow-up care are being transferred to primary care and locally where people live. Interprofessional collaboration among providers of different professional designations will be of increasing importance to optimizing primary care capacity in years to come. There is a call for further exploration of models of interprofessional collaboration that might be applicable in Norwegian primary care. The aim of this study was to explore experiences of interprofessional collaboration between primary care physicians and nurses working in primary care by applying an intervention for people with type 2 diabetes. Specifically, this study was designed to strengthen and gain deeper insight into interprofessional collaboration between primary care physicians and nurses in primary care settings.

**Methods:**

We applied Interpretive Description as a research strategy. The participants within this study were primary care physicians and nurses from four different primary care practices in the western and eastern parts of Norway. We used semi-structured telephone interviews for collecting the data between January and September 2021.

**Results:**

The analysis revealed two key features of the primary care physicians and the nurses experience with interprofessional collaboration in primary care practices. The first involved managing the influence of discrepancies in their expectations of IPC and the second involved becoming aware of the competence they developed that allowed for better complementarity consultation.

**Conclusions:**

This study indicates that interprofessional collaboration in primary care practice requires that primary care physicians and nurses clarify their expectations and, in turn, determine how flexible they can become in changing their usual primary care practices. Moreover, findings reveal that nurses and primary care physicians had discrepancies in expectations of how interprofessional collaboration should be carried out in primary care practice. However, both the nurses and primary care physicians appreciated the blending of complementary competencies and skills that facilitated a more collaborative care practice. They experienced that this interprofessional collaboration represented an essential quality improvement in the primary care services.

**Trial registration:**

The trial is registered 03/09/2019 in ClinicalTrials.gov (ID: NCT04076384).

## Background

Interprofessional collaboration (IPC) in primary care can be defined as an integrative cooperation of different healthcare professionals, blending complementary competences and skills, making possible the best use of resources [1]. IPC is seen as a key component for high quality practice in health services [2]. IPC depends not only on individual professional competencies, but also on how professionals who share common goals work together with patients, families, caregivers, and local communities to deliver the highest quality of care [2, 3]. Due to an aging population and thus an increased amount of patients with long-term conditions, the World Health Organization recently added the suggestion that more health tasks and follow-up care should be transferred to primary care [4].

As a result, primary care has gained a more central place in today’s health care services and patients who have been served in the acute or specialist care sector increasingly have access to services closer to where they live [5]. IPC follow-up in strengthened systems of primary care practice has become a promising strategy to improve quality outcomes. The concept of primary care covers a wide range of services to ensure people receive quality comprehensive care ranging from health promotion and prevention to treatment, rehabilitation, and palliative care [4].

For primary care physicians (PCPs), who provide continuous and coordinated primary care in many countries [6, 7], there is a trend in Norway and globally toward increased workload pressure [8, 9]. PCPs in Norway are worried that they will not be able to give people the healthcare service and care that is expected of them [9]. Non-communicable diseases are a worldwide health challenge. The follow-up of these patients can be time and resource consuming, and PCPs report limited time available to conduct this follow-up as well as fulfilling care tasks that are a hard priority [10, 11]. Therefore, IPC collaboration among providers of different professional designations will be of increasing importance to optimize primary care capacity in years to come [12].

Despite the growing recognition of the importance of collaborative approaches, as has been mandated in policy reforms in Norway and globally [5, 13–15], healthcare systems struggle to define and achieve these new forms of collaborative practice [16, 17]. One study found that it may be more challenging to implement IPC in primary care than in other settings as care roles are less well differentiated in primary care than they are in hospitals [18]. More specifically, barriers to IPC in primary care include lack of formal team member structures, leadership models, and common goals and visions for care [18].

In 2001, a PCP scheme was established in Norway which was supposed to guarantee all citizens the right to high quality primary care led by a specific PCP. The PCP scheme is a list-based system which aims to ensure healthcare services with high accessibility and continuity for all residents. The majority of Norwegians living with chronic diseases such as type 2 diabetes are cared for by PCPs in primary care [19]. The minority of PCPs employ registered nurses to take care of a broad range of work including counselling people with type 2 diabetes in diabetes management and healthy lifestyle. There is limited research on delegated independent tasks to nurses in general practice in Norway [20]. Such studies conducted in the Norwegian context are important because these nurses traditionally have had a less autonomous role in the care and follow-up of persons in general practice suffering from common chronic diseases such as diabetes compared to that of nurses in other Scandinavian countries [7]. There is a call for further exploration of models of IPC that might be applicable in Norwegian primary care [21]. The aim of this study was to explore the experiences of interprofessional collaboration between PCPs and nurses working in primary care by applying an intervention for people with type 2 diabetes in general practice. Specifically, this study was designed to strengthen and gain deeper insight into IPC between PCPs and nurses in primary care settings.

### The intervention

In Norway, the national guidelines for diabetes [19] state that different approaches to stimulate patient’s empowerment can be used. Guided Self-Determination (GSD) [22–25] is a stepwise approach to deliver counselling that empowers patients to enhance self-management in the context of chronic conditions such as diabetes. Self-management can be defined as “the ability of the individual, in conjunction with family, community, and healthcare professionals, to manage symptoms, treatments, lifestyle changes, and psychosocial, cultural, and spiritual consequences of health conditions” [26]. GSD was used by the nurses in this study as a counselling approach to provide person-centred healthcare and self-management. Previous research has shown that nurses who used the GSD approach in primary care experienced it as a constructive counselling method in stimulating patients’ reflections and motivation for diabetes management [27, 28].

GSD is a comprehensive concept that can be difficult to implement in full [27]. In this study, we modified and adapted standard GSD routines to design an interprofessional diabetes follow-up program in primary care practice. The intervention consisted of a stepwise approach with four interprofessional consultations over a period for 12 months. Reflection sheets and a guide to communication skills (mirroring, active listening and values clarification response) guided patients and nurses through a mutual reflection process, empowering the patient to become self-determined, achieve life skills and enhance skills in self-management living with type 2 diabetes. Between the consultations, the participants could fill in reflection sheets using their own words and drawings to express and reflect on their experiences and any difficulties with the management of their disease in daily life as well as to formulate behavioural goals and plans to achieve improved self-management. The nurses using GSD would capture these reflections in the structured consultations as well as report on them in the medical records after the initial consultations. In subsequent consultations, they used them to focus on challenges, goals, plans for action as well as other main concerns patients might have at that moment. The PCP would join the consultation when there was a need to address a strictly medical issue, or they would be involved after the consultation. The nurses reported from the consultation in a systematic and structured medical record which would be read and attended to by the PCP. The structured record comprised national guidelines for follow-up of people with type 2 diabetes as well as a framework for those at risk of developing type 2 diabetes. It incorporated and attended to the principles of the GSD method, thus comprising aspects such as challenges in your life, behavioural goals and plans to act on those goals.

## Methods

### Design

In this study we used Interpretive Description (ID) as an approach to guide us through the research process [29–31]. ID is an inductive, qualitative methodology and uses design techniques and elements borrowed from the social sciences toward consideration of applied questions arising from the work of the practice disciplines. In this way, it allows a focus on developing knowledge within a clinical context that can be relevant and useful in the applied health field [29].

### Participants

The participants within this study were PCPs and nurses from four different primary care practices in the western and eastern parts of Norway. Notably, other healthcare professionals were not present in these primary care practices. Using purposive sampling, we sent the invitation by email separately to the PCPs and nurses. The original plan was to include all health care professionals attending the intervention at the four primary care practices to capture their experiences in individual interviews. Unfortunately, due to the Covid-19 pandemic, only two thirds of the PCPs actually attended the intervention. Although the invitation was issued to ten PCPs and six nurses, because of a busy schedule due to other Covid-19 tasks, we could only recruit 7 PCPs and 6 nurses, The PCPs, three females and four males between the ages of 38 and 64 years, had worked in that role from four to thirty-two years,. The nurses, all females between the ages of 35 and 58 years, had worked from nine to 33 years. All of these nurses had experience working in a PCP practice for at least eight years and four were diabetes nurse specialists.

### Data collection

We used semi-structured telephone interviews for collecting the data between January and September 2021. The interviews were scheduled to last thirty to forty minutes, but some lasted up to ninety minutes because of the rich accounts that the interviewees had of their practice and their enthusiasm to share these insights. The semi-structured interview guide, developed by BCHK and MG, focused on themes related to our research aim (see Table 1). It served to loosely guide the interviews; however, additional prompts and clarifying questions were also used to obtain data that was as rich data as possible for both groups. Slight differences between the interview questions for nurse and physicians reflected our preliminary understanding of their distinct roles and responsibilities and our interest in obtaining rich insights; however, in both types of interviews, the focus on the study aim remained the same. We also encouraged the participants to bring forward other topics relevant to them during the interviews.

A retired researcher from Western Norway University of Applied Sciences who was not otherwise involved in the project was hired to conduct the interviews and to transcribe them verbatim. Although the lead researcher was a nurse with experience as in the primary health care system and as a qualitative interviewer, the interviewer had limited relevant experience prior to being trained for this project. BCHK, MG and BFO oversaw the interview process, reading the interviews repeatedly and taking notes during this process to get a sense of the evolving picture as the interviews progressed and to guide probing for more specific details in the interviews.

**Table 1:** Themes in the interview guide

**Physicians:** What is your work situation like usually? How are chronically ill patients followed up usually? How do you as a physician wish to follow-up patients with a risk of developing chronic disease or with manifest disease? Thoughts and reflections around counselling in your primary care practice?
Experiences with this study in your practice? What kind of experience do you have working interprofessional in this study? Did you experience that there were any changes in the follow-up of your patients who had consultations with a nurse? Did you experience any negative or positive factors? Is there anything we haven`t spoken about in this interview that you wish to address?
**Nurses**: What is your work situation like usually? How are chronically ill patients followed up usually by the nurses? What accommodations were made so you could participate in the study?
Experiences with this study in your practice? What kind of experience do you have working interprofessional in this study? Did you experience that there were any changes in the follow-up of your patients who had consultations with you as a nurse?
How did you experience the training in GSD as a counselling approach? How did you experience giving counselling with GSD to patients? What do you think about applying this interprofessional follow-up in the future? Did you experience any negative or positive factors?
Is there anything we haven`t spoken about in this interview that you wish to address?

### Analysis

ID provided the strategic guidance for the analysis process. We had in mind that the first author, who had been working as a diabetes-nurse specialist in a PCP practice for several years, would have some prior expectations of the data material when it comes to perceptions of the interprofessional follow-up in primary care. Therefore, BCHK, MG and BFO analysed the data separately and together as a team. We strove to be open-ended in our coding process, as well as using an inductive approach without overly focussing on the structure of the interview guide in the initial phase. In keeping with ID methodology, we used a constant comparison method when analysing the data. In the initial phase we used a broad-based coding, not labelling any patterns or themes initially to avoid premature closure when interpreting the data (see Table 2). Important issues and patterns were discussed and agreed on before we moved further. The preliminary patterns in data material were compared and discussed against different angles and interpretations. ST and KL participated in the analytic process before we reached a consensus on the final thematic structure and concluded the process.

**Table 2:** Illustration of analytic steps

1	Reading the transcripts repeatedly to become familiar with the data
2	Open-ended coding process, inductive approach in the initial phase
3	Using constant comparison method to compare the different patterns inbetween each interview
4	Broad-based coding to avoid premature closure
5	Independently coding in the initial phase before discussing the codes in the research group
6	A consideration of the explicit aims of the study
7	Considering the data set from multiple angles
8	Reaching a consensus on the final thematic structure and concluding the process

### Patient and public involvement

A patient representative contributed to discussing the relevance of the study objectives and justification for undertaking the research. In addition, the user representative has contributed to decisions on methods related to data collection. The user representative participated in the development of adequate written patient information for the study.

## Results

The analysis revealed two key features of the nurses and PCPs experience with IPC in primary care practices. The first involved managing the influence of discrepancies in their expectations of IPC and the second involved becoming aware of the competence they developed that allowed for better complementarity consultation.

### Manging the influence of discrepant expectations

From the outset, it was apparent that the experiences of the professionals involved in these interprofessional primary care practices were profoundly shaped by the differing expectations with which they entered the interprofessional follow-up. Although both nurses and PCPs held common goals for their patients, some of the aspects in which the expectations of nurses and PCPs differed had to do with understandings of how they should collaborate and how close an interprofessional collaboration should be in a primary care practice. They also differed with respect to the extent to which they believed a mutual documentation system in the primary care practice can fulfil an interprofessional collaboration in patient consultations and in terms of their expectations for the availability of the PCP during the day.

Some nurses expected to work more closely with the PCP around the patient’s situation and challenges than had been the practice with some PCPs. Because of this, they preferred face-to-face meetings for discussing issues related to the patient. One of the nurses expressed it this way:

*“I think that the primary care physicians and nurses must work more closely around the individual patient. That we simply see each other physically, and that we have a closer collaboration around our common patient.” (Nurse 3)*.

Other nurses nuanced their expectations of how close this interprofessional collaboration regarding the patient should be, arguing that individual assessment should be made if a specific matter was to be raised with the physician, as one nurse’s account illustrates:

*“It`s no need for the PCP to join the consultation every time. It must be an individual assessment when there are medical things that must be decided… to work interprofessional means that the nurse should assess when the PCP should contribute or not in the consultation, and that can change from time to time, depending on what the topic is.” (Nurse 5)*.

Several of the PCPs emphasized that they had learned that a common reporting system promoted collaboration and communication. They reported that, in addition to making individual patient assessments, they gained insight into the nurses’ assessments during their joint documentation system, which in turn resulted in a more comprehensive follow-up of the patient’s situation. This approach to IPC reflected the context of the work overload situation in primary care practices and the reality that the time schedule did not allow for a collaborative nurse and PCP consultation. One nurse described this challenge as follows:

*“The logistic problem here is that when we need a PCP for an assessment, the PCP already has an appointment with another patient. The PCP does not have an empty time schedule when I have my patients. The PCP must join into my consultation in between many of his own. Sometimes we just discuss the consultation with the PCP afterwards because we do not need an answer right there and then, and then we contact the patient.” (Nurse 5)*.

The quote above illustrates that the nurses expected and wished that the PCPs could be more available to collaborate. At the same time, they realized that it was not always possible and therefore moderated their expectations and found an alternative way to handle and resolve the situation where it would have been natural and important to collaborate more closely. The PCPs, for their part, found that the expectation of an immediate response from the nurses was stressful in a busy everyday practice as the following quote highlights:

*“It became a little stressful to suddenly be involved in something with a patient when I did not know that the patient was here at the clinic in a consultation at all…we should make a greater effort to make room and show it in the time schedule to the PCPs so that one knows what is going to happen during the day.” (PCP 3)*.

Another perspective within the IPC was the expectation from all the PCPs that the time schedule would be adhered to. Thus, as this quote exemplifies, the differential financial implications for the collaborative partners could become an added stressor:

*“By working in this way, patients get better, the consultations take longer time, but as I see it, it is important from a societal perspective. But it costs more to run the practice according to such a model and I hope that in the long run there will be both acceptance that these are important investments for the patients, and that it can lead to financially higher rates back to the doctor’s office.” (PCP 3)*.

### Developing awareness of complementary competencies

Both the PCPs and the nurses found that there was an exchange of skills and competence as this practice evolved and they experienced this to generate complementary and improved consultations. This competence exchange is illustrated in the following quote from one of the PCPs:

*“Many PCPs sit alone and that can lead to not getting all the new recommendations. So now we learn from each other, I learn from the nurses, and they learn from us physicians. I may find that it has become a little less to do for me, and then I have gained some more knowledge when I look at what the nurses do.” (PCP 2)*.

The GSD made the nurses’ competence more visible for many of the PCPs, allowing them to discover that the nurses had essential counselling competence with respect to lifestyle changes. Therefore, by referring patients to a nurse consultation based on the GSD approach, the PCPs experienced that the patients received useful and constructive counselling focusing on lifestyle change and health promotion, as one explained below:

*“I think it’s easier to have a nurse in front of me to go into that lifestyle change part. Maybe they are not as dependent on the relationship with the patient as the PCP is. An entire hour has been set aside for that type of conversation and guidance, and then they get to angle it and raise difficult things in a way so that the patient better understands the situation, and hopefully creates motivation to work with the problem. I am pretty sure that the patients through this GSD model to work by have gained a much greater understanding and better starting point to work with the challenges.” (PCP 2)*.

Both the nurses and the PCPs experienced that tailoring a standardized template in the medical record that the nurses could follow led to the consultations being more structured for all parties. Because the nurses experienced that they could place the patients in the centre of the consultations, they asked the patients questions about their life and living with a chronic condition or a risk of a chronic condition in which they had to take an active and conscious stance.

The PCPs experienced that this IPC represented an essential quality improvement in the primary care services. The PCPs emphasized that one important reason for this quality improvement was that, through the GSD intervention, they had become better acquainted with each other’s specialties and thus gained insight into the advantage of capitalizing on the slightly different approaches to the patient. It was important for the PCPs that the content of the interprofessional follow-up consultation for the patients was of medically sound professionalism. This to ensure that current clinical diabetes guidelines were fulfilled and also that the GSD method was performed according to the desired standard. That the interprofessional follow-up was designed to ensure a high standard of health care for patients with diabetes was expressed in this way;

*“When the nurse gets to know the patients, she performs her own consultations. So, this is very good for me as a PCP, because suddenly she finds out that the patient has not been checked by e.g. ophthalmologist for a long time…. we see that the patient is followed up in accordance with the guidelines.” (PCP 6)*.

Therefore, each profession contributed by complementing the other’s assessments and tasks. The patients thus received a follow-up that became an integral part of the team at the primary care practice. In this way, the IPC developed a synergy of professional knowledge.

## Discussion

The aim of this study was to explore the experience of interprofessional collaboration between primary care physicians and nurses by applying an intervention for people with type 2 diabetes in primary care practice. Our findings reveal that interprofessional collaboration (with the use of Guided Self-Determination in the intervention for patients with type 2 diabetes) was influenced by coming to understand that different health care professionals had entered the collaboration with discrepant expectations. Despite that, they could also see that the interprofessional collaboration had an important influence on their ability to recognize one another’s competence.

Our findings revealed that the nurses and PCPs showed discrepancies in expectations of how IPC should be carried out in primary care practice. Although change takes time, the nurses and PCPs experienced competence development with benefits in the quality of their relationship with patients. They pointed toward improvements of the health services that were considered an essential intended outcome of the interprofessional intervention. Working in an interprofessional manner in primary care practices may well enhance the follow-up of people living with a chronic condition [2, 12]. However, as we found in our study, there seemed to be discrepancies in the expectations of how to actually perform that interprofessional collaboration in practice. A recent review article [32] concluded that it is critical to clarify in advance the primary care team’s expectations about what their consultation and collaboration can and will provide. Our findings indicate that IPC in primary care practice requires that PCPs and nurses clarify their expectations and, in turn, determine how flexible they can become in changing their usual primary care practices. More specifically, it appears that discussing explicit expectations for each professional’s role and task is key for successful implementing of IPC. When it comes to collaborative meetings, it is interesting that the nurses called for more collaborative meetings with the PCPs, while the PCPs had a preference for the opposite. The communication between PCPs and nurses is emphasized as an essential part in an IPC but might be hindered by lack of familiarity of other team-members’ role, lack of trust in other allied healthcare professionals and minimal overlap in work schedules [15].

Many studies have reported that, in a busy day, with time and resource constraints, it is difficult to change working methods and routines [15]. However, a recent study from Austria among PCPs highlighted that there is a shift in reform when it comes to thinking positively about developing interprofessional work models [33]. In our study, implementing the GSD acted as a resource and driving force for the introduction of interprofessional collaboration. This finding is in accordance with other studies which have reported that promoting interprofessional collaboration also depended on providing effective tools [3]. An Australian study conducted in primary care settings found that a structured management tool in primary care, social cognitive therapy, can enhance the PCPs’ confidence and self-efficacy in managing obesity and thus improve the follow-up [34]. However, the discrepancies in the expectations of how to perform the interprofessional collaboration in practice might also be complicated by PCPs heavy workload due to organizational and structural changes of more health services being transferred to primary care. Recent reports confirm that the overwhelming workload is still increasing in primary care practices [8, 35, 36]. This general perception of time constraints may help explain how challenging it can be to change provider perception as to time efficiency. However, implementing a new intervention in a well-established health service takes time, and it might be difficult to see the longer-term benefits, both for the patient who receives continuous follow-up and for the nurses and PCPs who probably will experience a better utilization of resources and competencies. Having them look beyond the immediate moment to the longer-range benefit for the patients, including their need for ongoing consultation from PCPs over time in maintaining their health with a chronic condition, might be difficult. This seems a relevant underlying problem explaining their general lack of interest in participating in more frequently collaborative meetings with the primary care nurses.

Previous research has reported that fragmentation in the follow-up of chronic diseases in primary care is experienced as a challenge for the PCPs and also in respect to IPC [3]. It is shown that PCPs experience fragmentation and decreased continuity of care as an important challenge to their professional identity as PCPs and to their provision of a holistic approach [35]. We found on the basis of our study that the common medical record system may stimulate more collaborative meetings with nurses and therefore might play a role in moderating the experience of fragmentation for the PCPs in the follow-up of chronic diseases in primary care practice. Moreover, co-location for nurses and PCPs might also have a positive impact on the IPC. This speculation is consistent with findings from a study by Graue et al., who found that a co-location of healthcare professionals may facilitate an improved quality of healthcare for persons with complex needs [37]. Underestimating the importance of a co-location for healthcare professionals therefore becomes a hindrance to sharing standards of care and may impede primary care nurses’ perception of collaboration and support from the PCPs. A natural co-location in a primary care practice might facilitate an interprofessional collaboration between the PCPs and the nurses, and as Reeves et al. point out [15], effective communication lines between the collaborating members of a team contributes to improvements in patient care. In contrast to our findings, the review from Reeves et al. found that physicians prefer synchronous communication that allows for real-time dialogue with the collaborating team; from their perspective, it is electronic communication that hinders communication and thus generates ambiguity [15].

In this current study, both nurses and PCPs reported that they appreciated the blending complementary competences and skills, making possible a more collaborative care practice. However, previous studies have found that lack of clarity around practice scopes and different functions of collaborating healthcare professionals, as well as possible fear of loss of the professional identity, can be associated with a depreciation of other healthcare professionals` skillsets and contributions [38, 39]. In our study, one explanation for the favourable attitude towards IPC was the expressed value related to the exchange of competencies. Another reason was related to the fact that both PCPs and nurses experienced the collaboration as benefiting the patients. At first glance, this finding is not particularly surprising, but previous research paints a different picture. An overview study that identified barriers and facilitators on interprofessional collaboration in primary care [3] found that some professionals were concerned about the benefits of collaboration for their patients. This was particularly true regarding the collaboration between nurses and physicians, where physicians transferred tasks to nurses. In addition, it was also highlighted that PCPs believed that the involvement of other healthcare professionals in their patient’s care could endanger or even hinder, relational continuity, which is a basic tenet in primary health care services. This contradiction between findings can be interpreted in the light of our study that the PCPs had acquired knowledge about the nurses’ competence, and they appreciated how the patient was followed up. Other studies also support the idea that insight and knowledge about other healthcare professional’s competence is an important success factor in achieving interprofessional collaboration [3].

## Strengths and limitations

These findings can be seen as a contribution to understanding what hinders or facilitates an IPC in Norwegian primary care. context. Although this study provides us with a preliminary knowledge arising from some primary care practices and thus cannot shade light on all primary care contexts, we consider the findings to enrich us with insights that may have relevance for understanding primary care practices beyond our study setting. A strength of this study is that its participants reflected variation in profession, work experience, age and gender, thus providing us with a range of context and interpretation. Since the primary care practices participating were limited in number and we are still in the initial phase of working in an interprofessional manner across the primary care sector, future research will be needed to understand interprofessional in primary care in its maturity.

## Conclusion

This study reveals that nurses and primary care physicians enter these interprofessional primary care collaborations with discrepant expectations in how they should be carried out. In particular, the extent of the PCP’s misunderstanding of nursing competence in general prior to entering the interprofessional collaboration seems remarkable and worthy of further investigation in other jurisdictions. Clearly, clarifying these expectations can play a key role in influencing how flexible these health care providers can become in changing their usual primary care practices, and will facilitate integration of interprofessional collaboration in primary care practices. However, these findings also demonstrated that both nurses and physicians experienced the value of blending complementary competencies and skills, and appreciated engaging in a more collaborative care practice. From the perspective of the participants in this study, learning the skills of interprofessional collaboration represents a meaningful quality improvement opportunity in primary care services.

## Data Availability

The dataset used for the current study is available from the corresponding author on reasonable request.
